# Self‐Assembled Porphyrazine Nucleosides on DNA Templates: Highly Fluorescent Chromophore Arrays and Sizing Forensic Tandem Repeat Sequences

**DOI:** 10.1002/ejoc.201800683

**Published:** 2018-07-10

**Authors:** Mariia V. Ishutkina, Alice R. Berry, Rohanah Hussain, Olga G. Khelevina, Giuliano Siligardi, Eugen Stulz

**Affiliations:** ^1^ Department of Organic Chemistry Ivanovo State University of Chemistry and Technology Sheremetev Av. 7 RF‐153000 Ivanovo Russia; ^2^ School of Chemistry & Institute for Life Sciences University of Southampton Highfield SO17 1BJ Southampton UK; ^3^ Diamond Light Source Harwell Science and Innovation Campus Didcot OX11 0DE Oxfordshire UK

**Keywords:** Functional organic materials, Supramolecular chemistry, Fluorescence, Porphyrinoids

## Abstract

The formation of chromophore arrays using a DNA templating approach leads to the creation of supramolecular assemblies, where the optical properties of the overall system can be fine‐tuned to a large extent. In particular, porphyrin derivatives have been shown to be versatile building blocks; mostly covalent chemistry was used for embedding the units into DNA strands. Self‐assembly of porphyrin modified nucleosides, on the other hand, has not been investigated as a simplified approach. We report on the synthesis of a magnesium(II) tetraaza porphine (MgTAP) coupled to deoxyuridine, and array formation on DNA templates which contain well‐defined oligo(dA) segments showing strong fluorescence enhancement which is significantly larger than that with a Zn‐porphyrin. The use of the deep‐eutectic solvent glycholine is essential for successful assembly formation. The system allows for sizing of short tandem repeat markers with multiple adenosines, thus the concept could be adaptable to in vitro forensic DNA profiling with a suitable set of different chromophores on all nucleosides.

## Introduction

DNA has emerged to be a most versatile template in supramolecular chemistry due to formation of a predictable structure in form of the B‐DNA through sequence specific recognition of the complementary strand. The availability of tailor‐made nucleosides for automated solid support synthesis (SPS) of DNA has allowed to create well‐defined new functional molecules, and particularly the formation of multi‐chromophore arrays can be achieved in a programmable manner^1^ This concept is essential for the fundamental understanding of the interplay of the chromophores when placed in a pre‐determined three‐dimensional arrangement, which usually is in the major groove of the double stranded DNA (dsDNA). The minor groove has also been used to arrange optically active substituents.^2^ A number of substituents such as pyrenes,^3^ perylenes,^4^ porphyrins,[2b], 5 metal complexes,^6^ and nanoparticles^7^ have been investigated. The formation of helical chromophore stacks has revealed that strong electronic coupling, leading to efficient energy transfer systems, can be tuned using both the nature of the substituent and the underlying DNA sequence, which controls the distance between the units and leads to functional optoelectronic systems, for example for light harvesting, up‐conversion, hybridization probes, or photo‐responsive systems.^8^ It can therefore be expected that we will see an increasing use on DNA multichromophore arrays in biology, medicine and materials science.^9^

While those systems are perfectly well suited for the synthesis of diverse arrays due to the programmability of the sequence in SPS, the necessity to prepare a dimethoxy trityl (DMT) protected phosphoramidite building block also limits the availability of functionalities to some extent as they need to be compatible with the chemistry of SPS, and issues with coupling efficiency and purification may lead to low yields. The use of self‐assembly of nucleosides on DNA templates, on the other hand, is an attractive method to assemble longer arrays in a simplified approach as it relies solely on the recognition of the complementary base from a single stranded DNA template.^10^

Many investigations reported on the interactions of water soluble porphyrins with single and double stranded DNA, and non‐canonical DNA structures such as G‐quadruplexes, leading to either intercalated or stacked arrays, and the literature provides guidance to selectively obtain one over the other form of arrays.^11^ These are not the focus point of our investigations as we seek to create arrays of defined length, structure and composition, which requires making use of the hydrogen bonding pattern of the nucleobases. In this respect, Balaz and co‐workers have reported a diaminopurine functionalized porphyrin, which is able to recognize a thymidine base through complementary hydrogen bonding.^12^ Interestingly, here the helicity of the final array depended strongly on the annealing rate in the system where a poly(T) strand of variable length served as template: slow cooling led preferentially to right‐handed helices, but fast annealing gave left‐handed arrays.

Another related system described by Schenning et al. includes naphthalene and π‐conjugated oligo(*p*‐phenylene)‐vinylene,^13^ where a pH‐dependent switch between left‐ and right‐handed assembly was observed.^14^

Similarly, a nile red modified nucleoside reported by Varghese and Wagenknecht showed self‐assembly to form left‐handed helically twisted H‐type (aqueous solution) or J‐type (toluene) packing in nanovesicles, which is different to the right‐handed packing observed when covalently attached to a DNA template.^15^ The same group had shown previously that not only single chromophore nucleosides,^16^ but also mixtures of chromophores such as nile red and pyrene are accessible, which show exciton dissociation by electron transfer from a photo‐generated exciton on the chromophore stack to an appended fullerene.^17^ Other DNA‐induced chromophore aggregates were prepared using anthracene by Iizawa^18^ or cyanine dyes by Armitage.[2d]

## Results and Discussion

**Figure 1:**
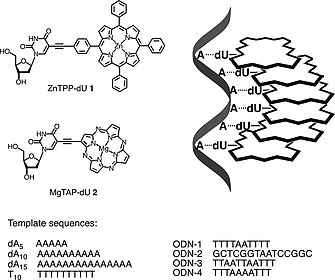
Structures of the porphyrin (**1**) and porphyrazine (**2**) nucleosides for self‐assembly on oligo(dA) template sequences, and schematic representation of the ODN templated assembly of chromophores.

Inspired by these reports, we explored the use of a zinc porphyrin 2′‐deoxyuridine building block (ZnTPP‐dU **1**, Figure 1) which serves us well for covalent DNA modification.^19^ In addition to the porphyrin, we also studied a new derivative based on magnesium(II) 5,10,15,20‐tetraaza porphine (porphyrazine, MgTAP‐dU **2**); TAP (and the related phthalocyanines) derivatives are promising molecules for use in photovoltaics,^20^ theranostics,^21^ photodynamic therapy,^22^ or as anti‐fibrillogenic agents.^23^ Both nucleosides **1** and **2** were tested for their ability to form stable non‐covalent arrays against adenosine containing ODN templates by virtue of natural base‐pairing, and their optical properties were analyzed using UV/Vis and fluorescence spectroscopy. Since **2** showed strong fluorescence signal enhancement upon complex formation, we also investigated this unit for its suitability to report on the length of forensically relevant short tandem repeat (STR) markers containing multiple adenosines.

The synthesis of **1** followed literature procedures[5f] using Sonogashira coupling^24^ between acetylene‐ZnTPP and 5‐iododeoxy uridine (5‐I‐dU) (for experimental details see electronic supporting information). Nucleoside **2** was synthesized by coupling a mono‐brominated MgTAP to 5‐ethynyl deoxyuridine^25^ through Sonogashira coupling. The MgTAP‐Br was obtained by bromination of MgTAP using NBS in ethanol.^26^ (On a side note, the successful Sonogashira coupling on the MgTAP‐Br also proves that the carbon carrying the bromide is sp^2^‐hybridised, which was not entirely clear before.^26^)

A challenge was to find a suitable solvent system for ODN binding as aqueous solvents could be ruled out due to the virtual insolubility of both **1** and **2** in water. Using DMSO or DMF as solvent (either neat or with added buffer) did not give any spectroscopic evidence that the building blocks would be assembling on any oligo(dA). However, recent reports by Hud et al. show that the deep eutectic solvent glycholine, which is composed of a 4:1 molar ratio of glycerol and choline chloride, supports the formation of DNA origami folding.^27^ We therefore focused on the use of this solvent system. While this will inevitably limit in vivo application, it is not an issue per se in supramolecular chemistry.

The absorbance and fluorescence spectra of **1** displayed the characteristic features of porphyrins in glycholine (Figure S1, see Supporting Information for full spectroscopic analysis): the main absorbance showed the B‐band at 423 nm and the Q‐bands at 560 nm and 601 nm, whereas the fluorescence spectrum showed two peaks at 605 and 659 nm with relative intensities of 1 and 0.51, respectively. (It should be noted that glycholine has a strong UV/Vis absorbance below 300 nm, therefore the relevant DNA part in the UV/Vis and CD spectra could not be analyzed in this solvent, and the data rely on the porphyrin and porphyrazine parts in the spectra.)

For **2**, the UV/Vis spectrum showed a strong absorbance at around 330 nm and a weaker broad absorbance at 604 nm, which is comparable to the MgTAPBr;^26^ the emission maximum was found at 428 nm (*λ*_ex_ = 330 nm). Temperature dependent measurements did not reveal any strong and extended aggregation of **1** as both absorbance and emission did not significantly change on cooling from 70 °C to 10 °C (Figure S1, S2; Table S1, S2); the same can be said for the absorbance of **2** (Figure S3; Table S3). However, for **2** a 1.4‐fold increase in fluorescence was observed, which indicates weak self‐aggregation at low temperatures (Figure S4; Table S4).

**Figure 2:**
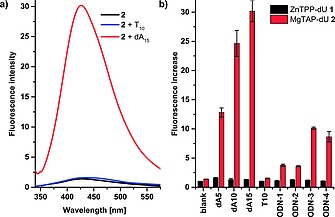
Left: representative example of fluorescence enhancement of **2** after binding to dA_15_, and showing no change upon addition of T_10_; fluorescence is normalized to **2**. Right: Fluorescence enhancements of both **1** and **2** on their own (blank), and when bound to different adenosine containing templates. For sequences see Figure 1.

Initially, we studied the self‐assembly of both building blocks on oligo(dA) sequences of variable lengths, namely dA_5_, dA_10_ and dA_15_. The UV/Vis spectra of 1:1 mixtures of dA*_n_* with both **1** and **2** (with respect to binding sites at 15 µm concentration of **1** or **2**) did hardly change upon slow annealing from 70 °C to 10 °C (Figure S1, S3; Table S1, S3). For the porphyrin **1**, the fluorescence spectra showed a small increase in fluorescence of a factor 1.6 ± 0.05 for the binding to dA_5_, and of about 1.3 for the binding to dA_10_ and dA_15_, indicating formation of a multichromophore assembly on the template strands (Figure 2, S2; Table S2). While we normally observe quenching of porphyrin emission when attached to DNA in multichromophore system,[5e], 28 the weak enhancement could be explained by shielding of the porphyrins from the highly polar solvent through π‐stacking along the ssDNA template.

In sharp contrast, the porphyrazine **2** showed significantly increased fluorescence with increasing length of the template (Figure 2, S4; Table S4). The fluorescence increase is (12.8 ± 0.8)‐fold for dA_5_, (24.6 ± 2.2)‐fold for dA_10_, and (30.2 ± 1.8)‐fold for dA_15_. The value for dA_15_ is about 15 % lower than would be expected when considering a linear increase, and the system therefore might show saturation when using longer templates. When corrected for the increase in fluorescence of **2** itself, the values are 9.1, 17.5 and 21.4 fold, respectively. Addition of T_10_ as template did not have any effect on the fluorescence of **2**, supporting that binding is governed by the complementary hydrogen bonding between dA and dU.

**Figure 3:**
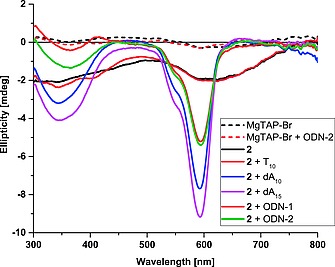
CD spectra of porphyrazine **2** demonstrating the formation of highly ordered arrays upon binding to adenosine containing template ODNs.

The array formation was also confirmed using synchrotron radiation CD spectroscopy (SRCD, Figure 3).^29^ A control MgTAP lacking the nucleoside moiety (e.g. MgTAP‐Br) did not show any CD signals; addition of ODN‐2 did not change the spectrum, thus the porphyrazine itself does not interact with DNA. MgTAP‐dU **2** on the other hand has two broad negative induced CD signals at 595 nm and at 330 nm, arising from chirality transfer of the attached nucleoside. Addition of T_10_ to **2** did not alter the CD spectrum, whereas dA_10_ and dA_15_ induced a significant sharpening and increased intensity of the negative signal at 595 nm, and also led to a more pronounced negative signal at 330 nm. This indicates highly ordered porphyrazine array formation along the oligo(dA) template with strong induction of chirality and extended π‐stacking. This array formation would explain the increase in fluorescence due to isolation of the chromophore from the highly polar solvent.

We next probed the response of **2** to ODN templates where di‐adenosine units were placed in different sequence context (Figure 2, S3, S4; Table S3, S4). Inserting an A_2_ unit within either an oligo‐T (**ODN‐1**), or within a random 15‐*mer* sequence (**ODN‐2**) gave rise to a corrected 2.7‐fold and 2.6‐fold increase in fluorescence, respectively. The similarity of the bis‐**2** adduct in **ODN‐1** and **ODN‐2** is also seen in the CD spectra which show identical peak position and intensity at 595 nm (Figure 3). Placing two separated dA_2_ units with an oligo‐T (**ODN‐3**) increases fluorescence by a factor of 7.3, while an dA_4_ unit (i.e. two adjacent dA_2_ units) increases it by a factor of 6.2. The response to **ODN‐4** is slightly lower than expected. Overall, this shows that **2** responds selectively to the specific number of repeating adenosines within a given sequence context; having the same number of repeating adenosines adjacent to each other gives a lower response than when they are separated.

The step‐wise increase of the fluorescence with increasing dA_2_ units could therefore be used to probe the number of dA*_n_* repeat sequences. This prospect is particularly intriguing for forensic DNA profiling, which relies on the analysis of short tandem repeat (STR) sequences in specific loci on the genome and thus assignment of specific alleles. Generally, STR typing is performed using PCR with fluorescence labelled primers, followed by electrophoretic separation of the STR allele and sizing against an STR ladder.^30^ Since this requires multiple steps in a specialized environment, we surmised that our system could report on the length of specific STRs using simple association and fluorescence readout. To test this hypothesis, we selected several representative loci from the European Standard Set of core STR loci^31^ and used model ODN sequences for binding with **2**.

**Figure 4:**
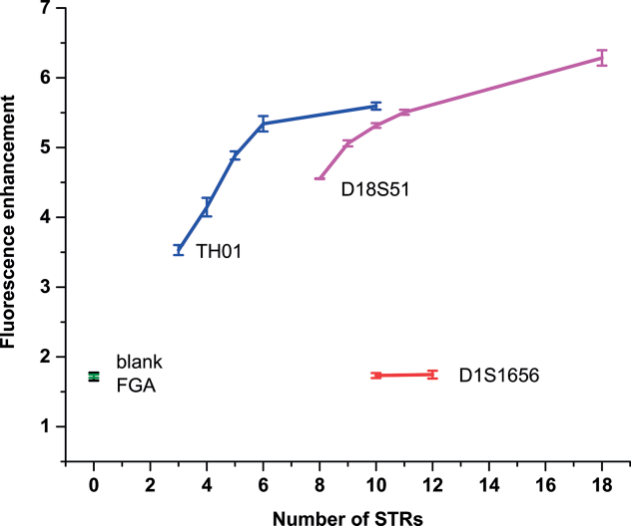
Sizing of forensic STRs by selective binding of MgTAP‐dU **2** to the target sequences. The number of repeat sequences can directly be correlated to the relative increase in fluorescence intensity. For sequences see Table 1.

To distinguish different sequence contexts and selectivity towards repeating dA*_n_* units, we chose the loci FGA (CTTT repeat) as negative control, D1S1656 (TAGA repeat), TH01 (AATG repeat), and D18S51 (GAAA repeat) (Table 1). For each STR locus we selected different alleles, i.e. increasing number of STRs, which span most of the biologically relevant lengths of the alleles. Addition of **2** to the sample ODNs showed that there is excellent selectivity towards STR markers that contain adjacent adenosines (Figure 4, S5; Table S5). For the control FGA, where no adenosines are present in the STR, no signal enhancement was detected. Similarly, the different alleles of D1S1656, which does not contain adjacent adenosines, did not give rise to a positive signal. For both FGA and D1S1656, the signal enhancement was equal to the blank sample (no DNA present). In contrast, analysis of the alleles of both TH01 and D18S51 gave significant signal enhancement, with increasing amplification corresponding to increasing length of the allele.

**Table 1:** Sequences of the short tandem repeat probes used for sizing the number of repeat units by fluorescence enhancement^a^

STR name	repeat unit	Sequence 5′–3′
		Alleles (*n*)
D1S1656	[TAGA]*_n_*[TGA]_0–1_ [TAGA]*_n_*[TAGG]_0–1_ [TG]_5_	CAACT [TAGA]*_n_* [TG]_5_ CTCTT
		*n* = 10, 12
TH01	AATG	ATTAT [AATG]*_n_* TAAGT
		*n* = 3, 4, 5, 6, 10
D18S51	GAAA	GCA AC [AGAA]_8_ AAAG A
		*n* = 8, 9, 10, 11, 18
FGA	CTTT	ACTCA [TTTC]_3_ TTTT TTCT [CTTT]_7_ CTCC [TTCC]_2_ ACTAT
		Allele 15

a Sequences are taken from MIST STR DNA Internet Data Base https://strbase.nist.gov/coreSTRs.htm.^30^

In the case of TH01, the number of STRs (*n*) ranges from *n* = 3 to 14 in forensic samples. For shorter alleles (*n* = 3 to 6, Table 1), the increase in signal was linear, and the number of STRs can directly be read out from the fluorescence intensity (Figure 4). For a longer allele (*n* = 10), the system seems to reach a saturation point. Therefore, the longer alleles used in profiling (*n* = 10 to 14) may not be readily distinguishable under the current conditions.

The locus D18S51 contains alleles with a much larger variability in numbers: the STRs range from *n* = 7 to 39 and represents a challenging STR locus. Overall, the signal enhancement was becoming linear with respect to higher STR numbers (n > 10). Nevertheless, a clear increase was detectable over a range of STRs with selected repeats ranging from 8 to 18, and the longer dA repeat unit (dA_3_ vs. dA_2_ in TH01) seems to prevent a saturation up to this level. Here, the number of STRs can directly be determined over the entire range of alleles.

## Conclusions

Overall, the porphyrazine is far better suited to create self‐assembled fluorescent chromophore arrays on a DNA template than the porphyrin; the porphyrin itself also forms arrays but does not lead to a significant change in optical properties. The eutectic solvent glycholine does not interfere with simple base‐pairing, shown by the selective formation of porphyrazine stacks on complementary adenosines in the template.

As few as two adjacent adenosines are sufficient to induce a significant increase in fluorescence of the porphyrazine. The stepwise increase in fluorescence upon increasing the number of adjacent adenosines allows for sizing the overall length of an oligo(dA) sequence through the readout of the fluorescence intensity. This concept is particularly intriguing for applications in analyzing DNA sequences which contain multiple repeats of short tri‐ or tetranucleotide units, which is used in allele identification. This forms the basis of forensic DNA analysis, and here we demonstrate that with our system the number of short tandem repeat sequences in selected core loci can directly be assessed. This approach would need to be tailored to other STR sequences by varying the probe nucleobase and the chromophores, where it could potentially be applied to microchip technology where the target DNA could be analyzed on surfaces by simple annealing and fluorescence readout.

## Experimental Section

For the binding experiments using dA*_n_*‐containing units, mixtures in glycholine (300 µL) were prepared to give a final concentration of 15 µm in **1** or **2**, and an ODN concentration of equal molarity in adenosine. The samples were heated to 40 °C under vacuum for several hours to remove the pyridine and water, and then transferred to a fluorescence cuvette at 40 °C. Variable temperature absorbance and fluorescence recordings were taken either from 70 °C to 10 °C at 10 °C intervals with 10 min equilibration time, or at 70 °C and 10 °C with cooling over 3 h. The enhancement was determined as a ratio of signal intensity I(10 °C)/I(70 °C). All measurements were performed in triplicate apart from **1** itself.

For analysis of STR sequences, samples were prepared and analyzed analogously with a final concentration of 2 µm of **2** and 0.02 µm of ODN in a final volume of 200 µL glycholine. The samples were annealed from 70 °C to 20 °C over the course of 3 hours, and the fluorescence spectra recorded at both temperatures. All experiments were performed in triplicate.

**Supporting Information** (see footnote on the first page of this article): Full details for synthesis and analysis are provided, and a full data set is available under https://eprints.soton.ac.uk/421225/.

## Supporting information

Supporting Information
