# Supplementary material for: Self‐Assembled Porphyrazine Nucleosides on DNA Templates: Highly Fluorescent Chromophore Arrays and Sizing Forensic Tandem Repeat Sequences
Source: European J Org Chem. 2018 Jul 10;2018(36):5054–9. doi: 10.1002/ejoc.201800683 (PMC6174987; doi:10.1002/ejoc.201800683)
Supplement: Supplementary file 1 — Supporting Information [file EJOC-2018-5054-s001.pdf]

*Eur. J. Org. Chem.* · ISSN 1099–0690

https://doi.org/10.1002/ejoc.201800683

# ***SUPPORTING INFORMATION***

***Title:*** Self-Assembled Porphyrazine Nucleosides on DNA Templates: Highly Fluorescent Chromophore Arrays and Sizing Forensic Tandem Repeat Sequences

***Author(s):*** Mariia V. Ishutkina, Alice R. Berry, Rohanah Hussain, Olga G. Khelevina,* Giuliano Siligardi, Eugen Stulz*

***Experimental:***

**Reagents and Materials**. The acetylene zinc-porphyrin,[1] MgTAPBr,[2] and 5-ethynyl-deoxyuridine[3] were prepared according to literature procedures. All chemicals and reagents were purchased from Sigma-Aldrich UK and used as received unless otherwise stated; 5-iodo-dU was obtained from Rasayan Inc, Encinitas CA, USA. Glycholine was prepared according to literature procedure.[4]

DNA strands for the templating were prepared in-house using solid phase synthesis (SPS) which followed standard phosphoramidite chemistry using an Applied Biosystems Expedite DNA synthesiser (1000 Å CPG) with reagents obtained from Tides Service, Haar, Germany (standard phosphoramidites, reagents, solvents).

$^{1}$H and $^{13}$C{$^{1}$H} NMR spectra were recorded at 400 MHz and 101 MHz, respectively, using a Bruker DPX400 spectrometer. UV-Vis spectra were recorded on a Varian Cary 300 Bio spectrophotometer using quartz cells (Hellma) with 1 cm path length. Fluorescence spectroscopy experiments were conducted using a Varian Cary Eclipse spectrophotometer using quartz cells (Hellma) with 1 cm path length. Excitation for fluorescence experiments was carried out at the sample's $\lambda_{max}$. The temperature was controlled using a Varian Cary Temperature Controller and peltier system with a Varian Cary Series II Temperature Probe. CD spectra were recorded at Diamond Light Source, Didcot UK, using beamline B23.[5] Samples were prepared analogously as for the UV-vis spectroscopy, and measured in quartz cuvettes, l = 3 mm (200 μL), step 1 nm, bandwidth 1 nm, time per point 0.5 s, 3 repeats.

**Synthesis of TPP-dU 1**

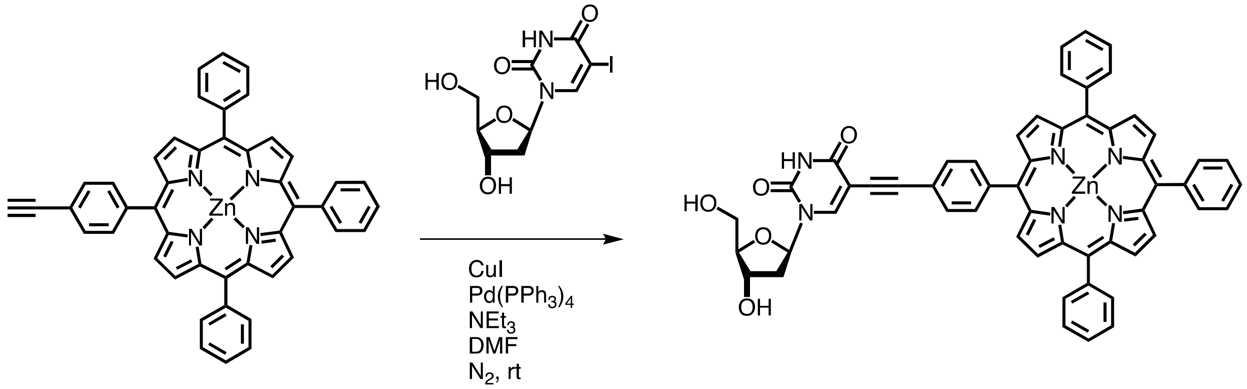


Zn(II)-5-*p*-ethynylphenyl-10,15,20-triphenylporphyrin (0.17 mmol, 82 mg), 5-iodo-2'-deoxyuridine (0.15mmol, 53.7 mg), CuI (0.06 mmol, 11.48 mg) and triethylamine (28.7 µL) were mixed in dry DMF (4.1 mL) in an oven dried, nitrogen purged flask containing molecular sieves. The reaction mixture was purged with nitrogen for 20 minutes before the addition of $Pd(PPh_3)_4$ (0.04 mmol, 50.6 mg). The reaction mixture was stirred in the dark under nitrogen for 24 hours, after which further portions of CuI (0.06 mmol, 11.5 mg) and $Pd(PPh_3)_4$ (0.04 mmol, 50 mg) were added, and the reaction was left for another 2 hours. After this time the reaction mixture was diluted with ethyl acetate (50 mL) and washed with a saturated solution of KCl (3 x 50 mL). The organic layer was dried with sodium sulphate and the solvent was removed in vacuo. The crude product was purified by column chromatography (silica, DCM/MeOH/EtOAc 100:0.5:0.5 → 100:2:2). The product was collected and dried under vacuum to produce 125 mg (0.134 mmol, 80 %) of **1** as purple solid.

$^1$H NMR (400 MHz, $CDCl_3$) δ ppm 8.94 (s, 2 H, β-pyrrole), 8.91 (s, 6 H, β-pyrrole), 8.43 (d, *J* = 8 Hz, 6 H, Ph-H), 8.31 (d, *J* = 8 Hz, 6 H, Ph-H), 8.17 (d, *J* = 8 Hz, 2 H, Ph-H), 8.03 (s, 1 H, N-H), 7.82 (d, *J* = 8 Hz, 2 H, Ph-H), 7.72 (s, 1 H, C(6)-H), 6.15 (t, *J* = 6 Hz, 1 H, C(1')-H), 4.39 (m, 1 H, C(3')-H), 3.85 (br s, 1 H, C(4')-H), 3.82 (d, *J* = 12 Hz, 1 H, C(5')-H), 3.73 (d, *J* = 12 Hz, 1 H, C(5')-H), 2.32 (m, 1 H, C(2')-H), 2.03 (m, 1 H, C(2')-H).

UV-vis: $CH_2Cl_2$ $\lambda_{max}$ nm (log ε) 231 (4.66), 313 (4.46), 406 (shoulder), 427 (5.63), 558 (4.25), 598 (3.89); glycholine 313 (shoulder), 406 (shoulder), 423 (5.47), 560 (4.12), 601 (3.16).

Fluorescence (glycholine) $\lambda_{ex}$ 423 nm, $\lambda_{em}$ nm (rel. int.) 605 (1), 659 (0.51)

MALDI-ToF MS (matrix α-Cyano-4-hydroxycinnamic acid) calcd. for $C_{55}H_{38}N_6O_5Zn$ m/z = 928.33, found 929.42.

**Synthesis of MgTAP-dU 2**

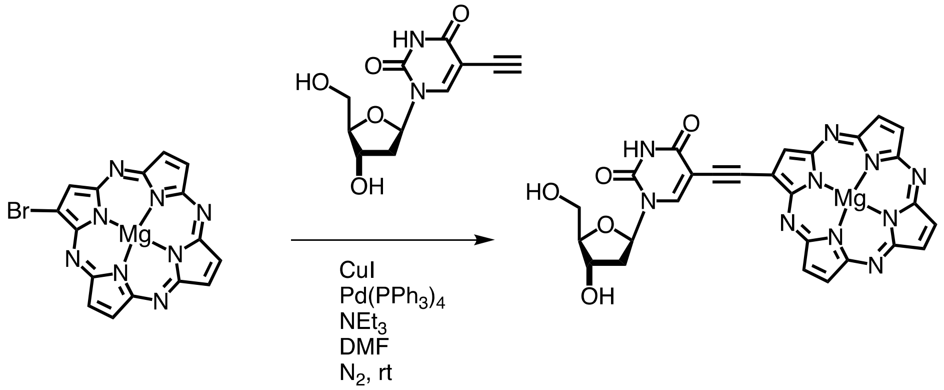


MgTAPBr (0.12 mmol, 50 mg), ethynyl-dU (0.16 mmol, 39.31 mg), CuI (0.04 mmol, 7.52 mg) and triethylamine (25 µL) were dissolved in DMF (5 mL) in an oven dried, nitrogen purged flask containing molecular sieves. This was flushed with argon for 20 mins before the addition of $Pd(PPh_3)_4$ (0.03 mmol, 34.65 mg). The reaction was stirred in the dark, under nitrogen for 24 hours. After 24 hours the reaction mixture was diluted with toluene, and the precipitated macrocyclic compounds were collected on a sintered filter. The solid mixture from the filter was dissolved in a toluene – THF mixture (1:1), and the solution was concentrated in vacuo. Purification was achieved by column chromatography (silica, toluene → 20 % THF in toluene) to give 15 mg (0.026 mmol, 21 %) of **2** as a dark blue solid of limited solubility in pyridine.

$^1$H NMR (500 MHz, pyridine-$d_5$) δ ppm 8.24 (s, 1 H, β-pyrrole), 7.37 (m, 6 H, β-pyrrole), 5.92 (m, 1 H, C(1')-H), 4.42 (m, 1 H, C(3')-H), 4.15 (m, 1 H, C(4')-H), 4.02 (m, 1 H, C(5')-H), 3.83 (m, 1 H, C(5')-H), 2.74 (m, 1 H, C(2')-H), 2.71 (m, 1 H, C(2')-H).

UV-vis: pyridine $\lambda_{max}$ nm (log ε) 325 (4.88), 610 (4.50); glycholine 604 (4.04).

Fluorescence (glycholine) $\lambda_{ex}$ 330 nm, $\lambda_{em}$ 428 nm.

MALDI-ToF MS (matrix α-Cyano-4-hydroxycinnamic acid) calcd. for $C_{45}H_{35}MgN_{10}O_7P$ (**2** + $H_2O$ + $P(O)Ph_3$) m/z = 883.12, found 882.2.

## Binding experiments of 1 and 2 with oligo-dA strands

For the experiments using $dA_5$, $dA_{10}$, $dA_{15}$, $T_{10}$, ODN-1 to ODN-4, mixtures in glycholine (300 μL) were prepared using stock solutions of ODNs in water (0.5 mM) and of **1** or **2** in pyridine (2.5 mM), to give a final concentration of 15 μM in **1** or **2**, and an ODN concentration of equal molarity in adenosine. The samples were heated to 40 ºC under vacuum for several hours to remove the pyridine and water, and then transferred to a fluorescence cuvette at 40 ºC. Variable temperature absorbance and fluorescence recordings were taken either from 70 ºC to 10 ºC at 10 ºC intervals with 10 min equilibration time, or at 70 ºC and 10 ºC with cooling over 3 h. The enhancement was determined as a ratio of $\lambda_{max}$(10 ºC) / $\lambda_{max}$(70 ºC). All measurements were performed in triplicate apart from **1**.

**Fig. S1:** Absorbance spectra of **1** in glycholine in absence and presence of oligo-dA strands.

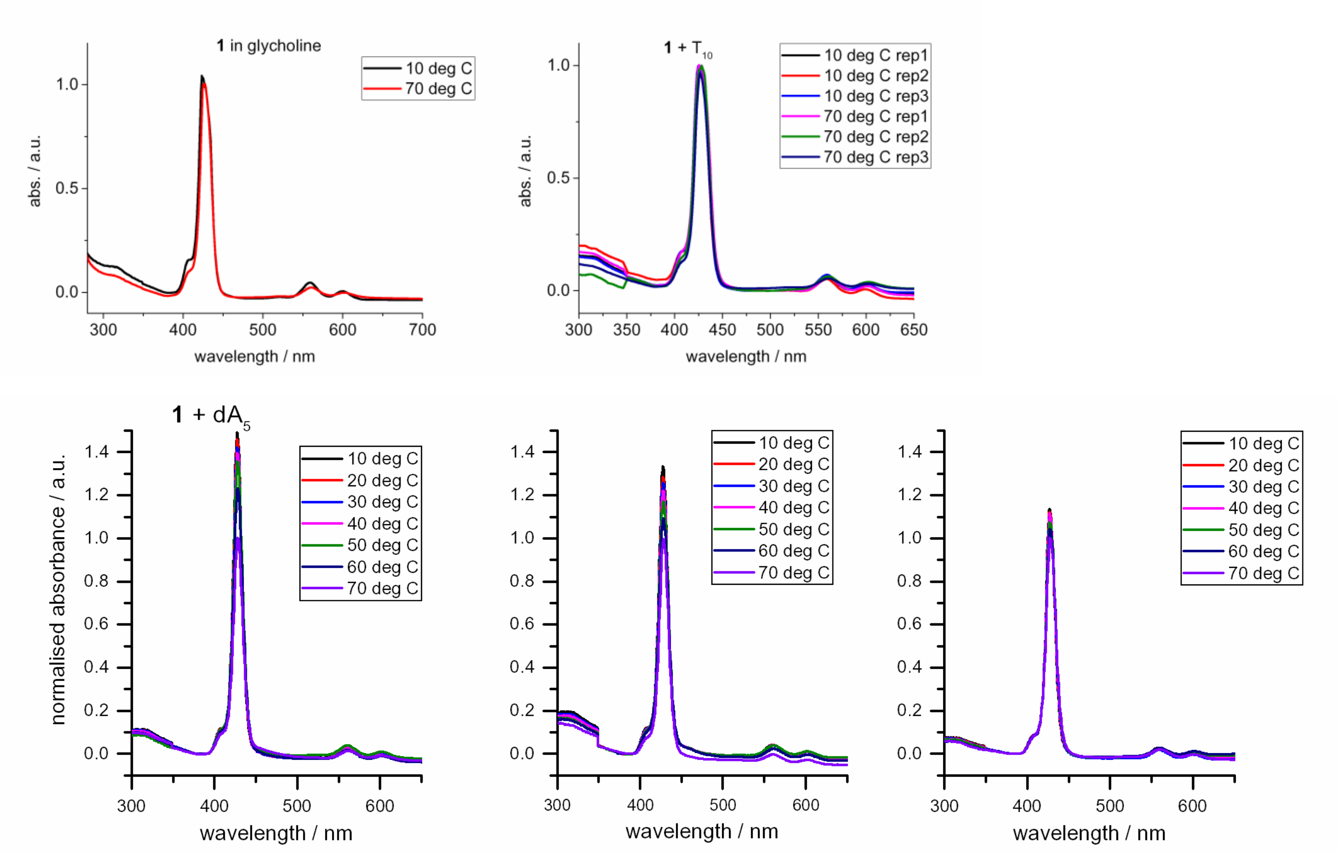

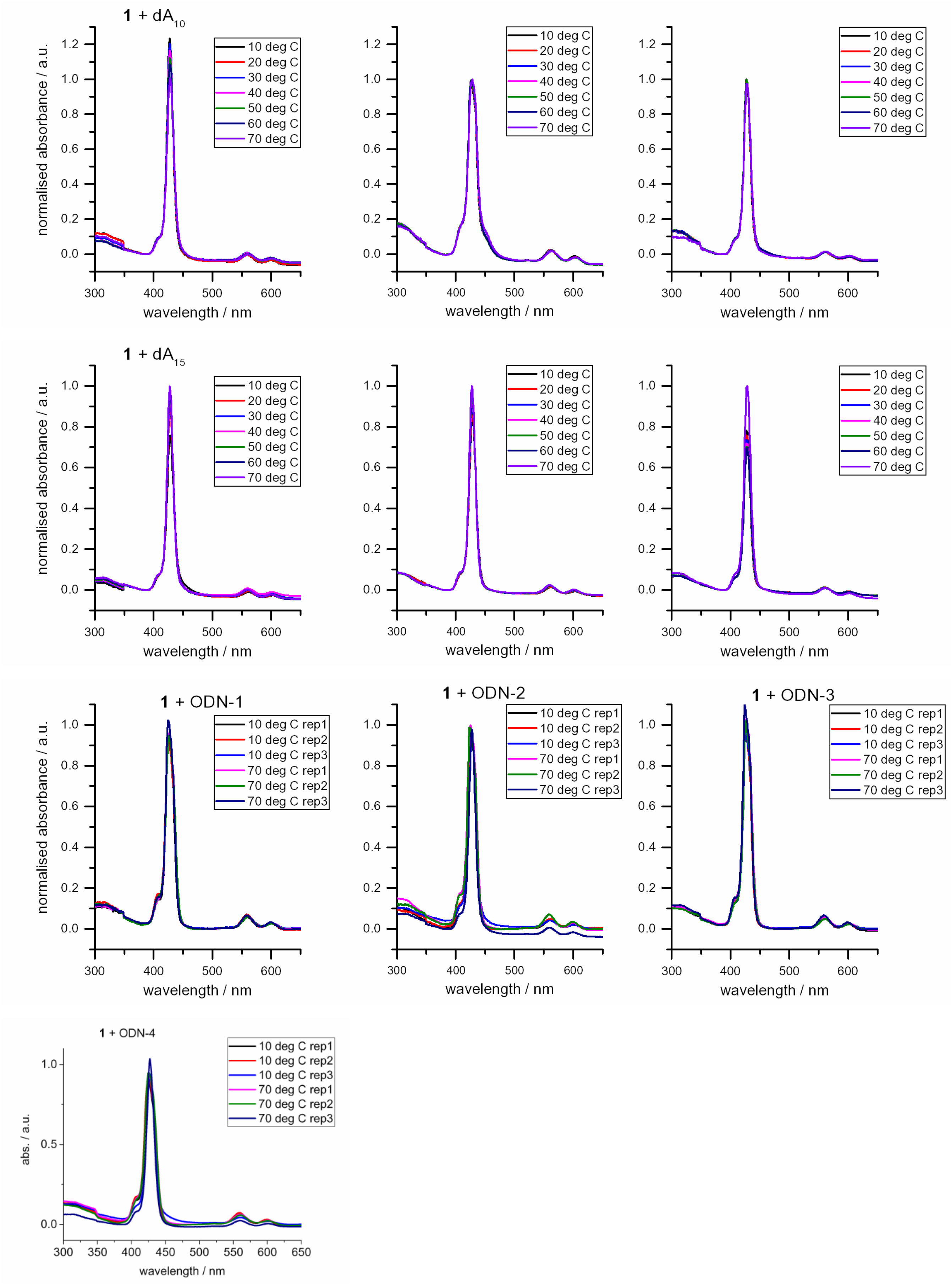

1 + dA10
1 + dA15
1 + ODN-1
1 + ODN-2
1 + ODN-3
1 + ODN-4
normalised absorbance / a.u.
abs. / a.u.
wavelength / nm
10 deg C
20 deg C
30 deg C
40 deg C
50 deg C
60 deg C
70 deg C
10 deg C rep1
10 deg C rep2
10 deg C rep3
70 deg C rep1
70 deg C rep2
70 deg C rep3

**Table S1:** Absorbance data for **1**.

| Sample | $\lambda_{max}$(10 ºC) / $\lambda_{max}$(70 ºC) | | | | |
|---|---|---|---|---|---|
| | repeat 1 | repeat 2 | repeat 3 | average | STD |
| **1** | 1.04 | | | 1.04 | ±0.00 |
| $A_5$ | 1.49 | 1.34 | 1.13 | 1.32 | ±0.18 |
| $A_{10}$ | 1.23 | 0.97 | 1.03 | 1.08 | ±0.14 |
| $A_{15}$ | 0.79 | 0.88 | 0.88 | 0.85 | ±0.05 |
| $T_{10}$ | 0.99 | 0.97 | 1.04 | 1.00 | ±0.03 |
| ODN1 | 0.98 | 0.99 | 0.92 | 0.96 | ±0.04 |
| ODN2 | 0.97 | 0.99 | 0.98 | 0.98 | ±0.01 |
| ODN3 | 0.98 | 0.98 | 0.89 | 0.95 | ±0.05 |
| ODN4 | 0.97 | 0.96 | 0.88 | 0.94 | ±0.05 |

**Fig. S2:** Fluorescence spectra of **1** in glycholine in absence and presence of oligo-dA strands.

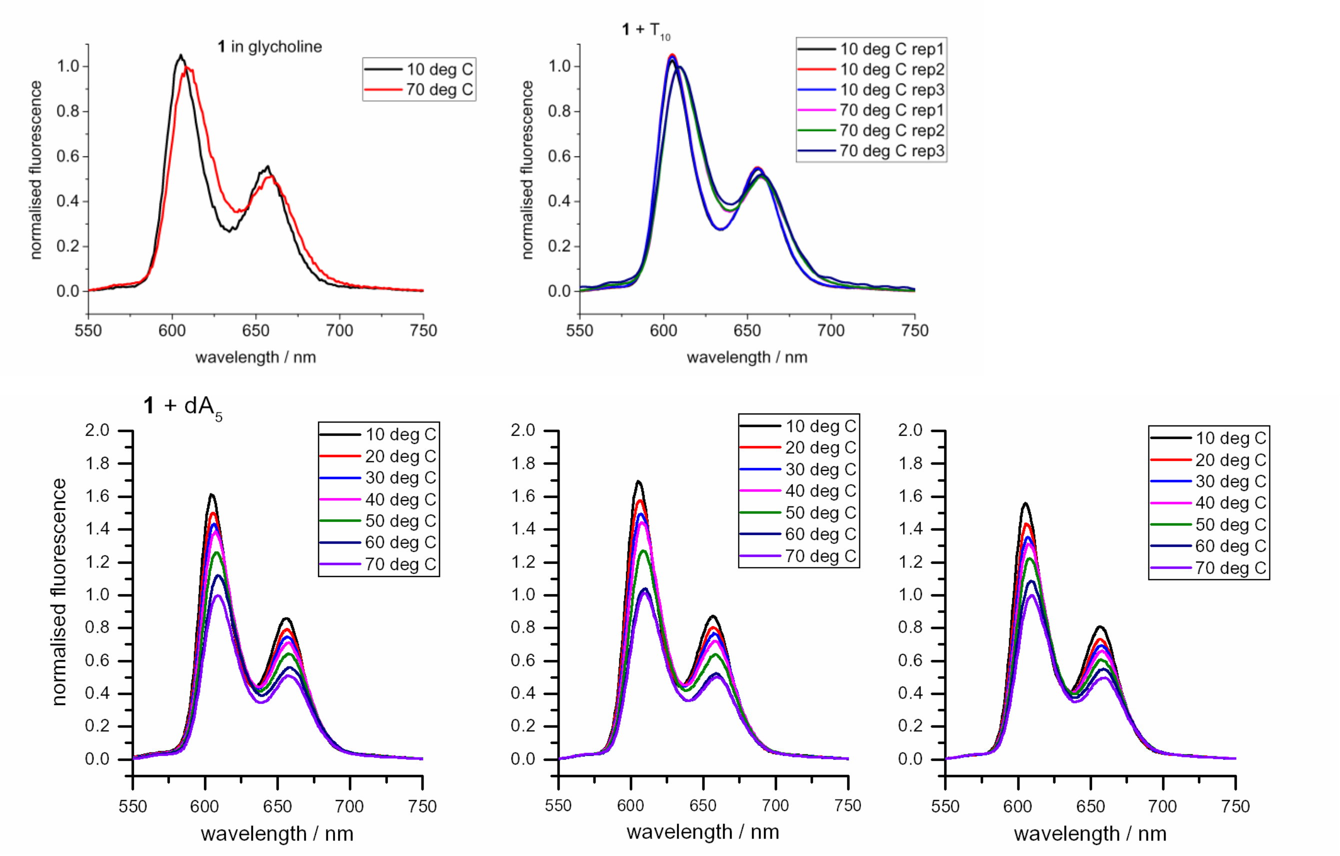

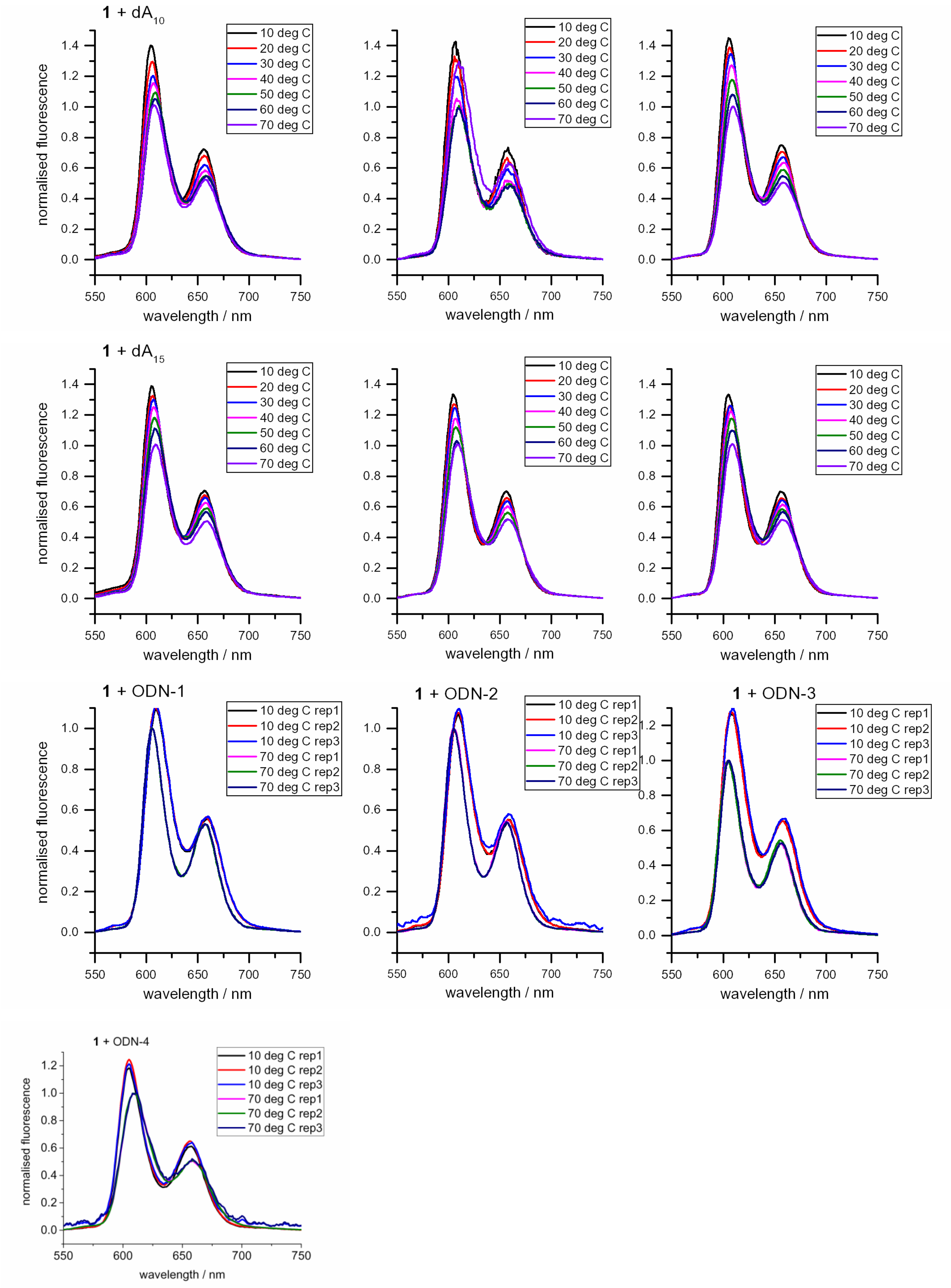

1 + dA10
1 + dA15
1 + ODN-1
1 + ODN-2
1 + ODN-3
1 + ODN-4
10 deg C
20 deg C
30 deg C
40 deg C
50 deg C
60 deg C
70 deg C
10 deg C rep1
10 deg C rep2
10 deg C rep3
70 deg C rep1
70 deg C rep2
70 deg C rep3
normalised fluorescence
wavelength / nm

**Table S2:** Fluorescence data for **1**.

| Sample | $\lambda_{max}$(10 ºC) / $\lambda_{max}$(70 ºC) | | | | |
|---|---|---|---|---|---|
| | repeat 1 | repeat 2 | repeat 3 | average | STD |
| **1** | 1.05 | | | 1.05 | ±0.00 |
| $A_5$ | 1.61 | 1.67 | 1.57 | 1.62 | ±0.05 |
| $A_{10}$ | 1.39 | 1.10 | 1.44 | 1.31 | ±0.18 |
| $A_{15}$ | 1.38 | 1.32 | 1.32 | 1.34 | ±0.03 |
| $T_{10}$ | 1.03 | 1.06 | 1.05 | 1.04 | ±0.01 |
| ODN1 | 1.10 | 1.10 | 1.16 | 1.12 | ±0.04 |
| ODN2 | 1.07 | 1.08 | 1.10 | 1.08 | ±0.02 |
| ODN3 | 1.27 | 1.28 | 1.32 | 1.29 | ±0.03 |
| ODN4 | 1.18 | 1.25 | 1.21 | 1.22 | ±0.03 |

**Fig. S3:** Absorbance spectra of **2** in glycholine in absence and presence of oligo-dA strands.

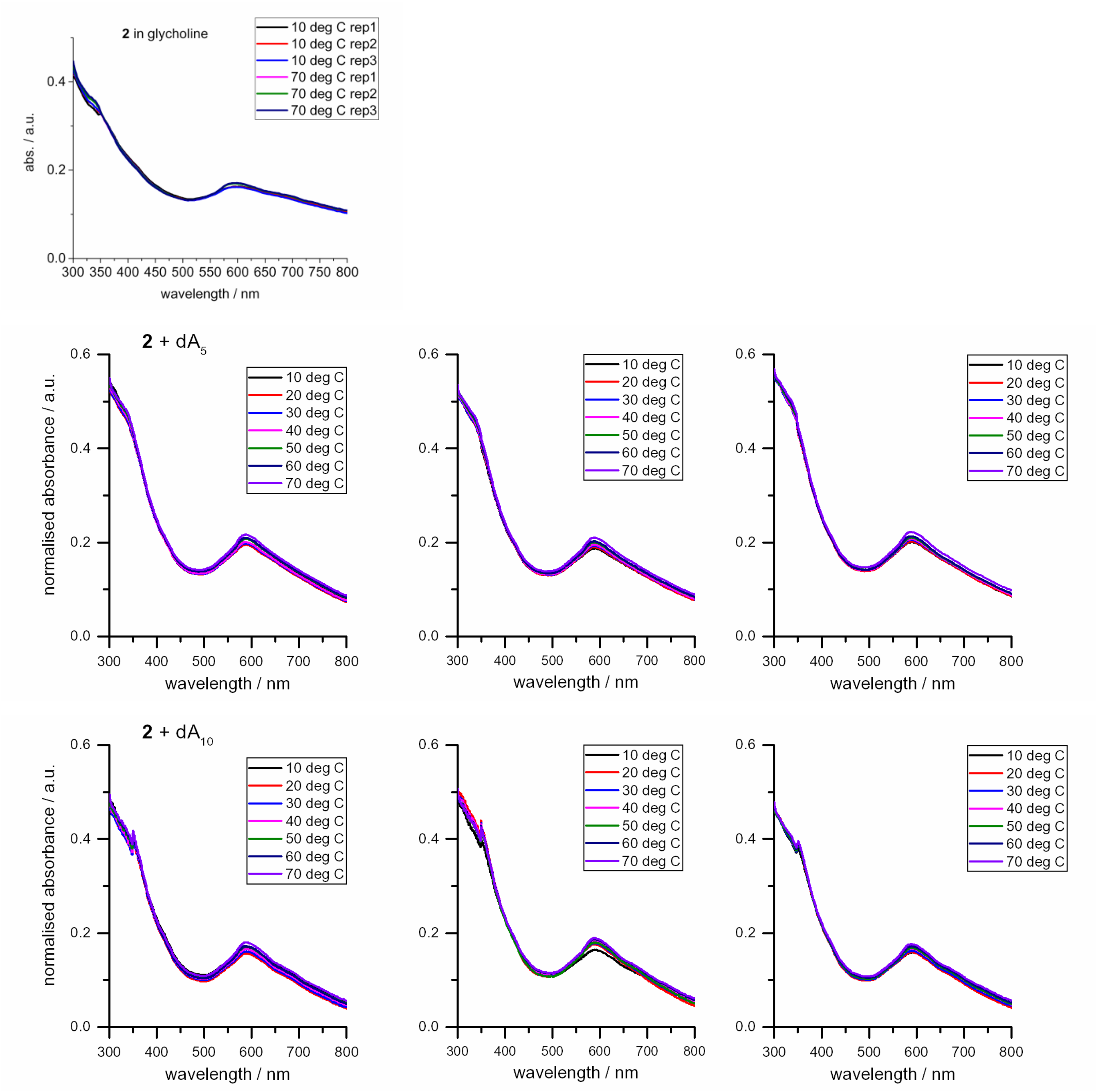

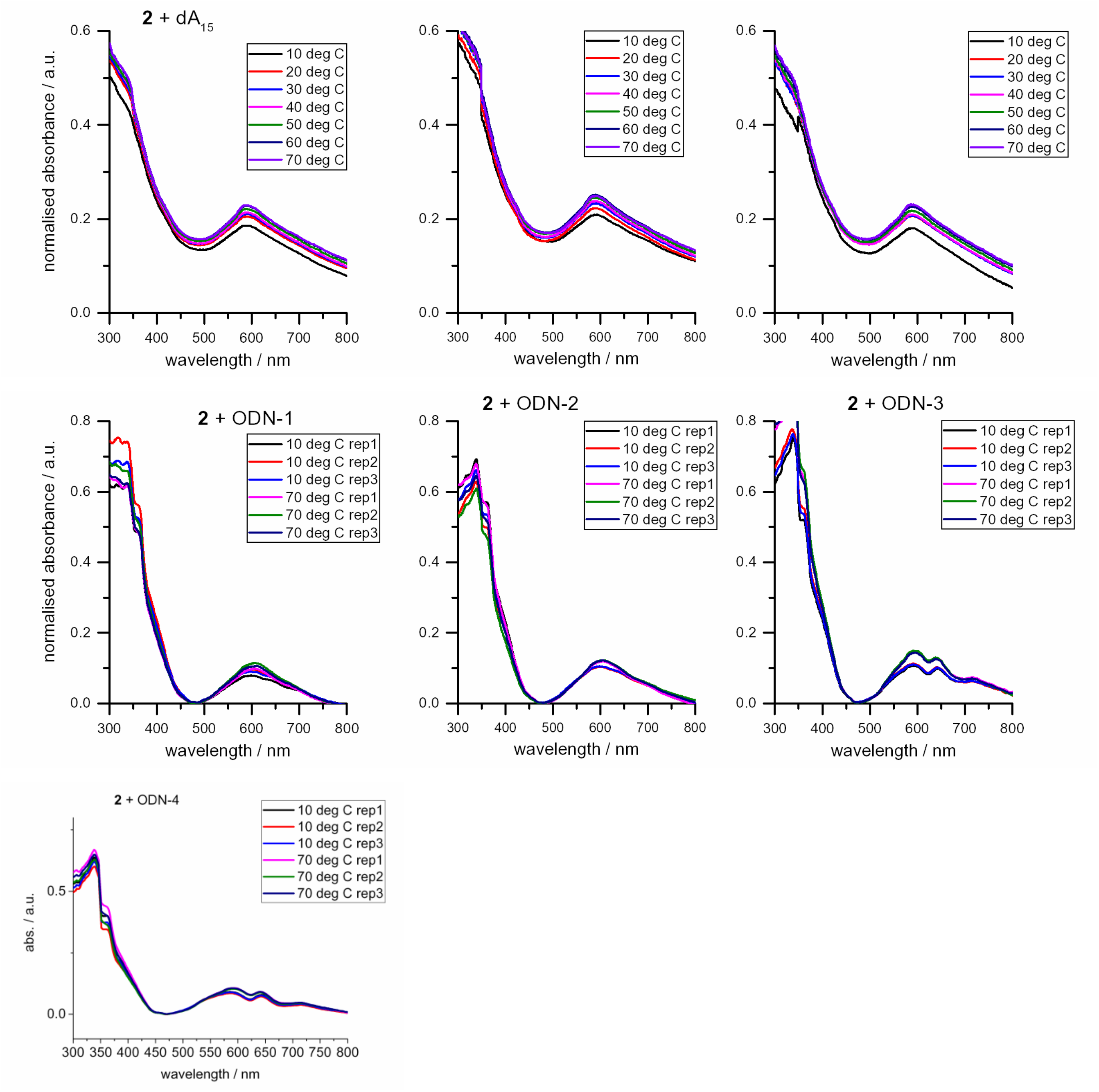

2 + dA15
normalised absorbance / a.u.
wavelength / nm
10 deg C
20 deg C
30 deg C
40 deg C
50 deg C
60 deg C
70 deg C
2 + ODN-1
2 + ODN-2
2 + ODN-3
10 deg C rep1
10 deg C rep2
10 deg C rep3
70 deg C rep1
70 deg C rep2
70 deg C rep3
2 + ODN-4
abs. / a.u.

**Table S3:** Absorbance data for **2**.

| Sample | $\lambda_{max}$(10 ºC) / $\lambda_{max}$(70 ºC) | | | | |
|---|---|---|---|---|---|
| | repeat 1 | repeat 2 | repeat 3 | average | STD |
| **2** | 0.96 | 0.96 | 0.95 | 0.96 | ±0.01 |
| $A_5$ | 0.91 | 0.89 | 0.90 | 0.90 | ±0.01 |
| $A_{10}$ | 0.93 | 0.86 | 0.90 | 0.90 | ±0.03 |
| $A_{15}$ | 0.81 | 0.84 | 0.78 | 0.81 | ±0.03 |
| $T_{10}$ | 0.93 | 0.90 | 0.96 | 0.93 | ±0.03 |
| ODN1 | 0.80 | 0.84 | 0.84 | 0.83 | ±0.02 |
| ODN2 | 0.88 | 0.84 | 0.86 | 0.86 | ±0.02 |
| ODN3 | 0.73 | 0.76 | 0.77 | 0.75 | ±0.02 |
| ODN4 | 0.85 | 0.83 | 0.84 | 0.84 | ±0.01 |

**Fig. S4:** Fluorescence spectra of **2** in glycholine in absence and presence of oligo-dA strands.

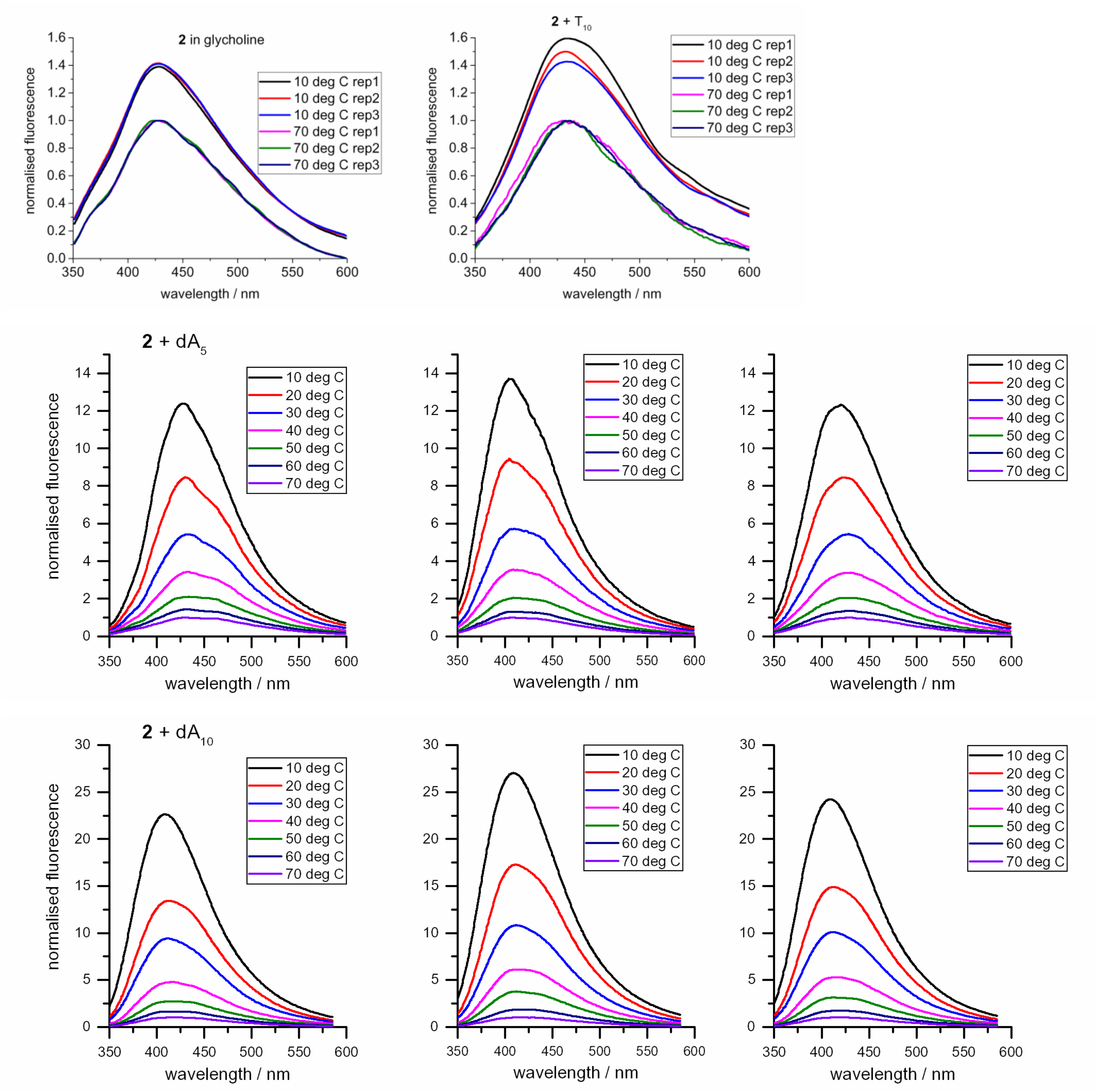

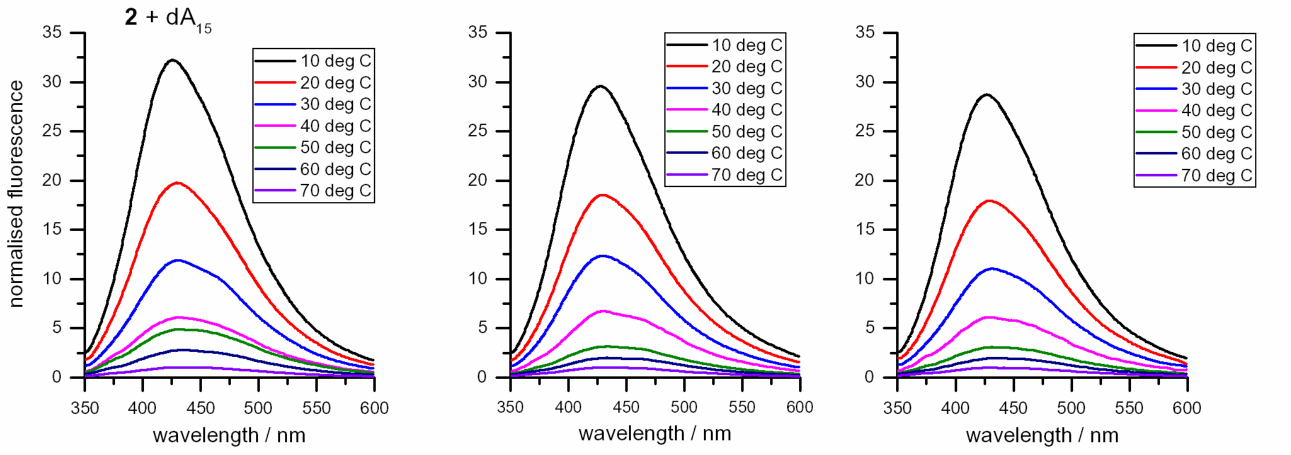
2 + dA15
normalised fluorescence
wavelength / nm
10 deg C
20 deg C
30 deg C
40 deg C
50 deg C
60 deg C
70 deg C

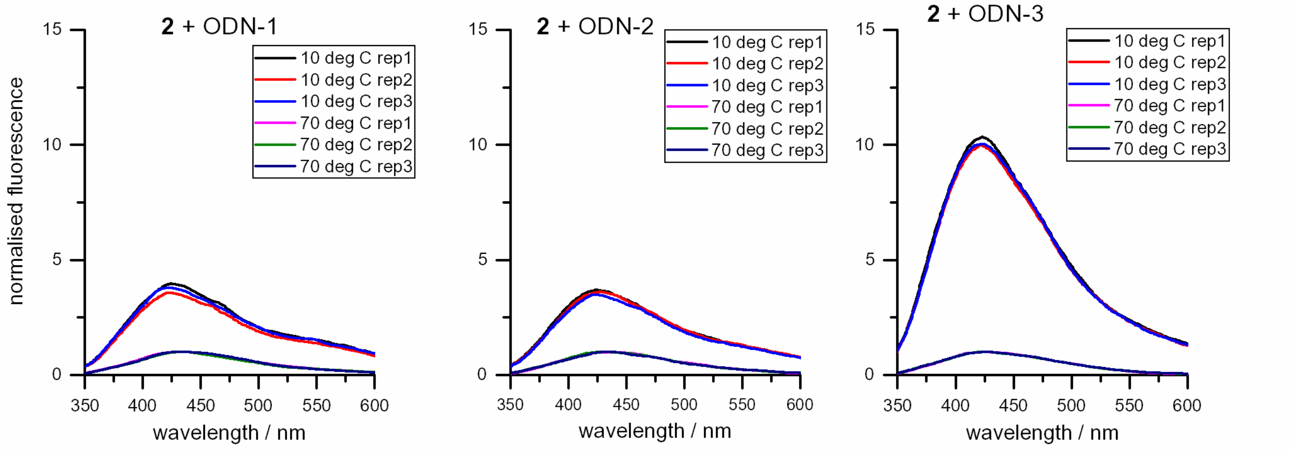
2 + ODN-1
2 + ODN-2
2 + ODN-3
normalised fluorescence
wavelength / nm
10 deg C rep1
10 deg C rep2
10 deg C rep3
70 deg C rep1
70 deg C rep2
70 deg C rep3

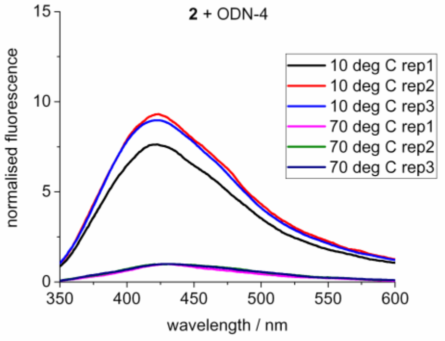
2 + ODN-4
normalised fluorescence
wavelength / nm
10 deg C rep1
10 deg C rep2
10 deg C rep3
70 deg C rep1
70 deg C rep2
70 deg C rep3

**Table S4:** Fluorescence data for **2**.

| Sample | $\lambda_{max}$(10 ºC) / $\lambda_{max}$(70 ºC) | | | | | |
|---|---|---|---|---|---|---|
| | repeat 1 | repeat 2 | repeat 3 | average | STD | corrected for **2** |
| **2** | 1.39 | 1.42 | 1.41 | 1.41 | ±0.01 | |
| $A_5$ | 12.39 | 13.71 | 12.32 | 12.81 | ±0.78 | 9.08 |
| $A_{10}$ | 22.64 | 27.03 | 24.24 | 24.64 | ±2.22 | 17.47 |
| $A_{15}$ | 32.22 | 29.58 | 28.73 | 30.18 | ±1.82 | 21.40 |
| $T_{10}$ | 1.60 | 1.50 | 1.43 | 1.51 | ±0.08 | 1.07 |
| ODN1 | 3.96 | 3.58 | 3.80 | 3.78 | ±0.19 | 2.68 |
| ODN2 | 3.69 | 3.61 | 3.48 | 3.60 | ±0.11 | 2.55 |
| ODN3 | 10.35 | 9.99 | 10.03 | 10.12 | ±0.20 | 7.18 |
| ODN4 | 7.64 | 9.32 | 8.98 | 8.65 | ±0.89 | 6.13 |

**Analysis of STR sequences in forensic core loci ODNs**

Samples were prepared analogously to the experiments with the oligo-dA sequences with a final concentration of 2 μM of **2** and 0.02 μM of ODN in a final volume of 200 μL glycholine. The samples were annealed from 70 ºC to 20 ºC over the course of 3 hours, and the fluorescence spectra recorded at both temperatures. The enhancement was determined as a ratio of $\lambda_{max}$(10 ºC) / $\lambda_{max}$(70 ºC). The instrument detector settings were adjusted for the different samples in order to gain maximum sensitivity but avoiding detector saturation. All experiments were performed in triplicate.

**Table S5:** Fluorescence data for **2** in the analysis of STR sequence lengths.

| STR name Allele used | repeat | sequence 5' – 3' | $\lambda_{max}$(10 ºC) / $\lambda_{max}$(70 ºC) | | | | |
|---|---|---|---|---|---|---|---|
| | | | rep. 1 | rep. 2 | rep. 3 | average | STD |
| **2** | | | 1.65 | 1.75 | 1.75 | 1.72 | ±0.06 |
| **D1S1656** Allele 10, 12 | $[TAGA]_n$ $[TGA]_{0-1}$ $[TAGA]_n$ $[TAGG]_{0-1}$ $[TG]_5$ | CAACT $[TAGA]_{10}[TG]_5$ CTCTT | 1.69 | 1.74 | 1.77 | 1.73 | ±0.04 |
| | | CAACT $[TAGA]_{12}[TG]_5$ CTCTT | 1.69 | 1.74 | 1.81 | 1.75 | ±0.06 |
| **TH01** Allele 3, 4, 5, 6, 10 | AATG | ATTAT $[AATG]_3$ TAAGT | 3.48 | 3.51 | 3.61 | 3.53 | ±0.07 |
| | | ATTAT $[AATG]_4$ TAAGT | 4.02 | 4.14 | 4.28 | 4.15 | ±0.13 |
| | | ATTAT $[AATG]_5$ TAAGT | 4.82 | 4.90 | 4.94 | 4.89 | ±0.06 |
| | | ATTAT $[AATG]_6$ TAAGT | 5.23 | 5.34 | 5.45 | 5.34 | ±0.11 |
| | | ATTAT $[AATG]_{10}$ TAAGT | 5.56 | 5.57 | 5.65 | 5.59 | ±0.05 |
| **D18S51** Allele 8, 9, 10, 11, 18 | GAAA | GCA AC $[AGAA]_8$ AAAG A | 4.55 | 4.56 | 4.55 | 4.55 | ±0.01 |
| | | GCA AC $[AGAA]_9$ AAAG A | 5.01 | 5.07 | 5.10 | 5.06 | ±0.04 |
| | | GCA AC $[AGAA]_{10}$ AAAG A | 5.33 | 5.28 | 5.34 | 5.32 | ±0.03 |
| | | GCA AC $[AGAA]_{11}$ AAAG A | 5.55 | 5.48 | 5.49 | 5.51 | ±0.04 |
| | | GCA AC $[AGAA]_{18}$ AAAG A | 6.41 | 6.22 | 6.22 | 6.28 | ±0.11 |
| **FGA** Allele 15 | CTTT | ACTCA $[TTTC]_3$ TTTT TTCT$[CTTT]_7$ CTCC $[TTCC]_2$ ACTAT | 1.70 | 1.73 | 1.74 | 1.72 | ±0.02 |

**Fig. S5:** Fluorescence spectra of **2** in glycholine in absence and presence of STR ODN sequences.

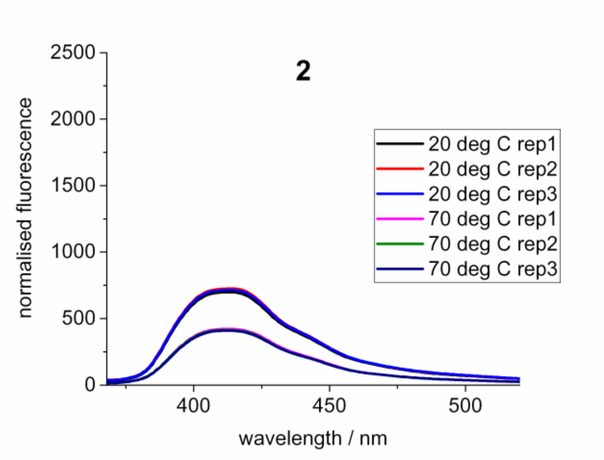


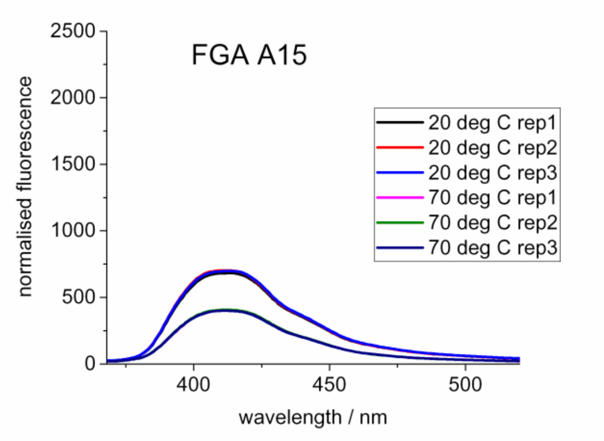

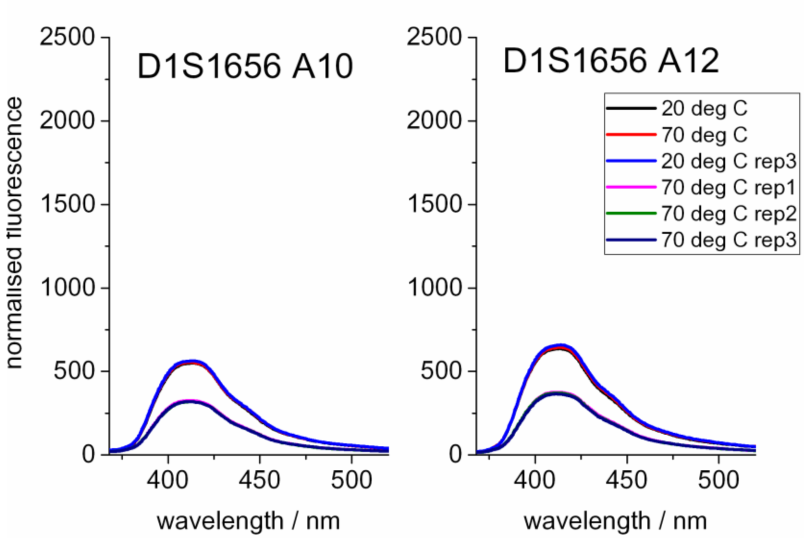
D1S1656 A10
D1S1656 A12
normalised fluorescence
wavelength / nm
20 deg C
70 deg C
20 deg C rep3
70 deg C rep1
70 deg C rep2
70 deg C rep3
2500
2000
1500
1000
500
0
400
450
500

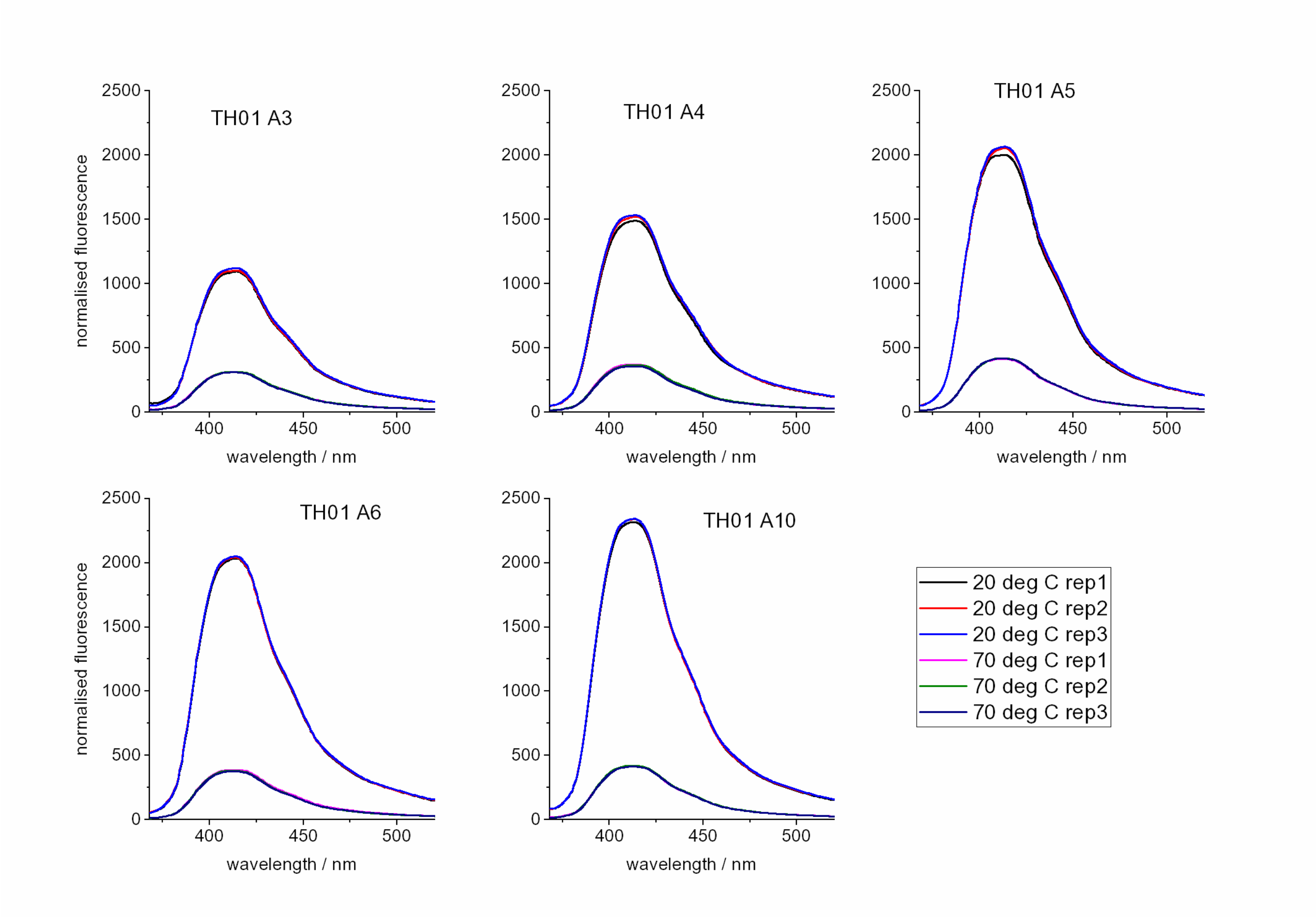
TH01 A3
TH01 A4
TH01 A5
TH01 A6
TH01 A10
normalised fluorescence
wavelength / nm
20 deg C rep1
20 deg C rep2
20 deg C rep3
70 deg C rep1
70 deg C rep2
70 deg C rep3
2500
2000
1500
1000
500
0
400
450
500

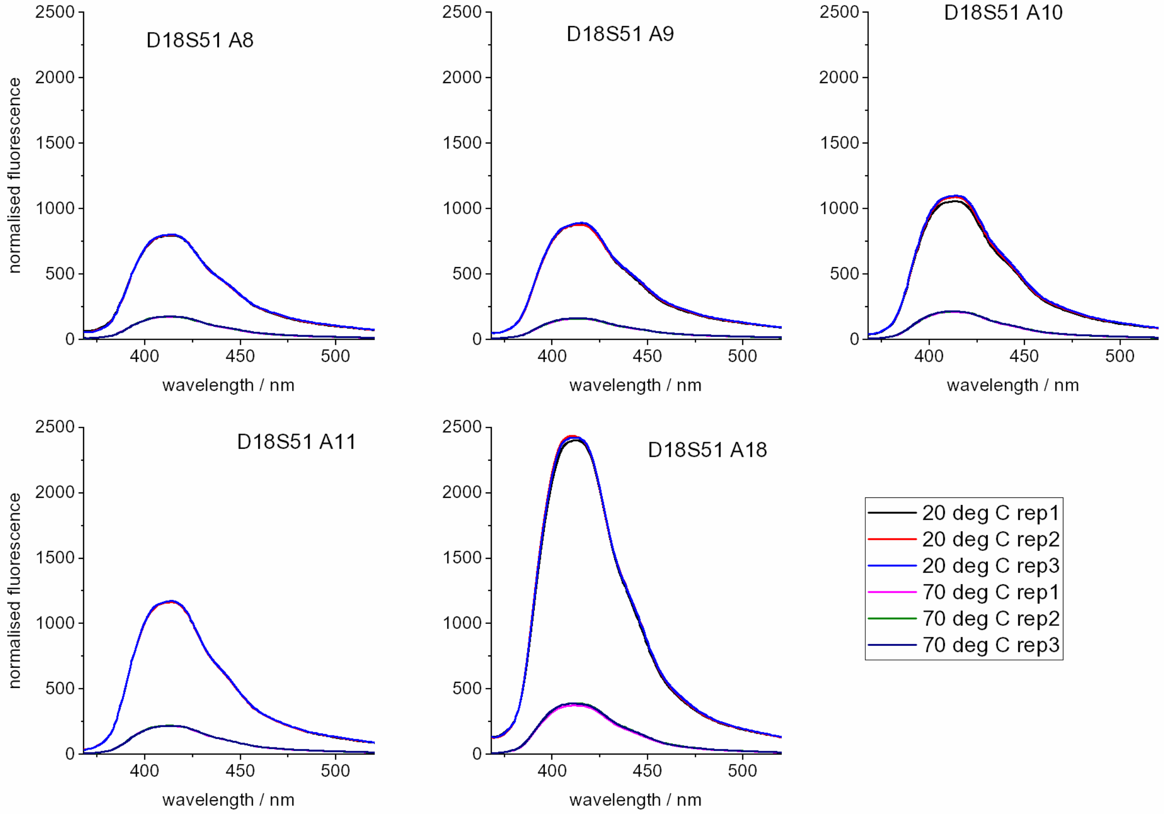


## References:


[1] I. Bouamaied, L. A. Fendt, D. Häussinger, M. Wiesner, S. Thöni, N. Amiot, E. Stulz, *Nucleos Nucleot Nucl* **2007**, *26*, 1533-1538.

[2] M. V. Ishutkina, O. G. Khelevina, E. N. Krylov, V. V. Aleksandriiskii, O. I. Koifman, *Russ. J. Organ. Chem.* **2015**, *51*, 1652-1658.

[3] S. Seo, K. Onizuka, C. Nishioka, E. Takahashi, S. Tsuneda, H. Abe, Y. Ito, *Org. Biomol. Chem.* **2015**, *13*, 4589-4595.

[4] I. Gallego, M. A. Grover, N. V. Hud, *Angew. Chem. Int. Ed.* **2015**, *54*, 6765-6769.

[5] a) R. Hussain, T. S. Jávorfi, G. Siligardi, *J. Synchrot. Radiat.* **2012**, *19*, 132-135; b) R. Hussain, T. Jávorfi, G. Siligardi, in *Comprehensive Chirality, Vol. 8*, Elsevier, **2012**, pp. 438-448.
